# Consumer Experiences and Perceptions of Genetic Counseling in Provider‐Mediated Genetic Testing

**DOI:** 10.1002/jgc4.70257

**Published:** 2026-07-24

**Authors:** Katherine A. Lauro, Bob Wong, Jason Iuliano, Margaret Peo, Jacqueline Park, Karin M. Dent, Angela R. Bradbury, Madison K. Kilbride

**Affiliations:** ^1^ Graduate Program in Genetic Counseling University of Utah Salt Lake City Utah USA; ^2^ College of Nursing University of Utah Salt Lake City Utah USA; ^3^ S.J. Quinney College of Law University of Utah Salt Lake City Utah USA; ^4^ College of Engineering University of Utah Salt Lake City Utah USA; ^5^ Department of Pediatrics University of Utah Salt Lake City Utah USA; ^6^ Department of Medical Ethics and Health Policy University of Pennsylvania Philadelphia Pennsylvania USA; ^7^ Division of Hematology‐Oncology, Abramson Cancer Center University of Pennsylvania Philadelphia Pennsylvania USA; ^8^ Department of Philosophy University of Utah Salt Lake City Utah USA

**Keywords:** attitudes, cancer, cardiovascular, experiences, genetic counseling, genetic counselor, genetic test results, genetic testing, physician‐mediated genetic testing, qualitative research

## Abstract

Provider‐mediated genetic testing (PM‐GT) is a hybrid model in which consumers initiate testing online, but a physician, typically from a third‐party network, is required to order the test. While genetic counseling is a critical component of result interpretation and medical decision‐making, its integration into PM‐GT remains limited. This study explored how consumers engaged with genetic counseling in the context of PM‐GT, including factors that influenced whether they pursued it and their perceptions of its value. Participants had previously undergone a 59‐gene panel test for cancer and cardiovascular disease risk through Color Health, a PM‐GT company. Twenty‐nine participants completed qualitative interviews about their testing experience and a follow‐up survey that was focused on genetic counseling. Post‐test counseling was required only for individuals with positive results and was optional for those with negative or variant of uncertain significance (VUS) results. Using quantitative content analysis and qualitative inductive content analysis, we found that while most participants did not engage in pre‐ or post‐test counseling, nearly half of those who had not yet spoken with a genetic counselor after receiving their results expressed interest in doing so in the future. Participants often viewed the counseling that was offered by the testing company as helpful for understanding their results, clarifying disease risk, and guiding medical decisions. Time constraints were the most frequently cited barrier to accessing genetic counseling services. These findings indicate a gap between consumers' interest in counseling and their actual uptake of services, suggesting that many consumers who could benefit from counseling do not receive it. More proactive integration of genetic counseling into the PM‐GT process could help bridge this gap and ensure that consumers are appropriately supported regardless of their test results.

## Abstract (highlights)

**What Is Known About This Topic:**

Provider‐mediated genetic testing (PM‐GT) allows consumers to initiate testing online, but a physician must authorize the order. Genetic counseling is essential for helping individuals interpret results and make informed health decisions, but is often limited in PM‐GT. Currently, little is known about how consumers use or perceive counseling in this setting.

**What This Paper Adds to the Topic:**

This study explores how consumers use and perceive genetic counseling in PM‐GT. Findings reveal a disconnect between consumers' interest in counseling and their actual use of services, suggesting opportunities to improve access and delivery.

## 1 Introduction

Consumer genetic testing has fundamentally transformed how individuals access their genetic information. To date, over 100 million people have undergone direct‐to‐consumer (DTC) genetic testing, a trend driven in recent years by declining costs and growing public interest in personal genomics (Henry 2021; Kilbride and Bradbury 2020). The process typically begins with individuals purchasing a test online and receiving a saliva sample collection kit from the testing company. Consumers then mail their DNA sample to the laboratory, and results are returned through an online portal (Shah and Domchek 2020).

In the traditional DTC model, companies market tests as a way for individuals to gain insights into ancestry, personal traits, and health‐related information (Majumder et al. 2021). This type of testing has historically screened for single nucleotide polymorphisms (SNP), which are single‐base‐pair substitutions that occur throughout the genome. However, SNP‐based technologies capture only a small fraction of genetic predispositions to disease (Bellcross et al. 2012). For example, 23andMe, a leading DTC genetic testing company, currently uses this testing to screen for 44 pathogenic variants in the *BRCA1* and *BRCA2* genes, which are linked to an increased risk of breast and ovarian cancer (23andMe, n.d.).

Over the past decade, a new model of consumer genetic testing has emerged—provider‐mediated genetic testing (PM‐GT). This model combines features of traditional DTC and clinic‐based testing. As with DTC testing, consumers still initiate the testing process online. However, PM‐GT differs in that it requires a physician's order, typically from a third‐party provider who does not directly interact with the consumer (Jiang et al. 2023; Majumder et al. 2021; Phillips et al. 2019). PM‐GT services typically use next‐generation sequencing (NGS), a high‐throughput technology that rapidly analyzes large portions of the genome (Majumder et al. 2021). These tests often focus on medically actionable conditions, such as cancer and cardiovascular disease syndromes, that were previously testable only through clinical settings (Color Health, n.d.). Whereas 23andMe's SNP‐based testing is limited to 44 *BRCA1* and *BRCA2* variants, NGS has greater than 99% sensitivity for identifying more than 3000 known pathogenic variants in these genes (Color Health, n.d.; LaDuca et al. 2020; Rebbeck et al. 2018; Truty et al. 2019). Given its capability, PM‐GT enables consumers to access more clinically meaningful genetic information than was previously possible through traditional DTC offerings.

Despite its importance in helping individuals interpret results and make informed health decisions, genetic counseling is not routinely integrated into consumer genetic testing. In both DTC genetic testing and PM‐GT, consumers are not required to consult with a genetic counselor (GC) before ordering a test. The traditional DTC model also does not require post‐test genetic counseling, regardless of the result. In contrast, some companies offering PM‐GT, such as Color Health, require consumers to speak with a GC only if they receive a positive result. Consumers who receive negative or variant of uncertain significance (VUS) results are not required to consult with a GC, though post‐test counseling is available if they choose to initiate it (Color Health, n.d.; Middleton et al. 2017).

The American College of Medical Genetics and Genomics (ACMG) and the National Society of Genetic Counselors (NSGC) issued position statements on DTC genetic testing in 2016 and 2019, respectively. Both organizations emphasize that a genetics professional should be available to help consumers decide whether testing is appropriate and to interpret results, particularly in the context of an individual's personal and family medical history (ACMG Board of Directors 2016; NSGC 2019). ACMG also highlights a number of potential risks associated with DTC genetic testing, including inadequate informed consent, inappropriate test selection, misinterpretation of results, and lack of knowledge about appropriate follow‐up care that can be mitigated by involving a board‐certified genetics professional, such as a GC, in the testing process (ACMG Board of Directors 2016).

Previous studies have examined the consumer experience with traditional DTC genetic testing and explored perceptions of the role of GCs in this space (Carere et al. 2016; Kilbride et al. 2024; Marzulla et al. 2021). Some have found that consumers report lower self‐efficacy and negative emotional responses after receiving DTC results (Carere et al. 2016; Kilbride et al. 2024). Another study showed that individuals who underwent genetic counseling after receiving DTC results sought help interpreting their findings in the context of their personal and family health history, particularly to understand potential medical actionability (Marzulla et al. 2021). Together, these findings point to unmet needs in the traditional DTC genetic testing space that genetic counseling could help address. However, little is known about consumers' experiences and perceptions of genetic counseling in the PM‐GT setting. To address this gap, the present study examined consumers' use of genetic counseling, the factors that influenced whether they spoke with a GC after receiving results, including barriers to access, and their attitudes toward the role and value of genetic counseling in the PM‐GT process.

## 2 Methods

### 2.1 Participants

This study was approved by the University of Utah Institutional Review Board. Participants were recruited from among individuals who had previously enrolled in the ENLITE Study (**E** valuating the Be **N** efits and **LI** mitations of the Next Generation of Consumer Genetic **TE** sting) at the University of Utah (K01 HG010903: Kilbride). The ENLITE study followed individuals who purchased and underwent Color Health's physician‐mediated genetic test for cancer and cardiovascular disease risk and measured a range of patient‐reported outcomes across three surveys.

Initial ENLITE Study recruitment was conducted through Research Match, a nonprofit program funded by the National Institutes of Health that uses non‐random sampling to enable investigators to identify self‐registered volunteers who are interested in participating in research studies for which they qualify (Harris et al. 2012). Potential participants were invited via email to complete an eligibility screener and provide informed consent. To be eligible for the ENLITE study, participants had to be at least 18 years old, be able to communicate in English by phone, be willing to purchase the Color Hereditary Disease Risk Test out‐of‐pocket, and not have previously undergone genetic testing for cancer or cardiovascular disease risk. Approached individuals were able to opt out of the study by not completing the eligibility screener.

### 2.2 Procedures

Between March and July 2023, 105 participants purchased Color Health's genetic test, which analyzed 59 genes associated with cancer and cardiovascular disease risk and included a pharmacogenomics report. Forty‐three eligible participants were invited to take part in a semi‐structured qualitative interview about their testing experience; 31 completed interviews between November 2023 and August 2024. Participant selection for interviews involved oversampling those with positive and VUS results to ensure that they were included in the sample population, as these individuals often face more complex counseling needs and medical decision‐making compared to those with negative results. Interviews were conducted by a female undergraduate research assistant enrolled at the University of Utah who received training on how to conduct qualitative interviews and used a structured interview guide (Author JP). Participants had no established relationship with the interviewer but were given information regarding her educational background at the start of the interview. Only the interviewer and interviewee were present during the interview. Interview questions explored participants' recall of their genetic test results, motivations for pursuing testing, post‐test experiences, behavioral performance and intentions following the receipt of their results, and overall attitudes toward testing. For participants whose test reports indicated a VUS result, the interviewer explicitly referenced that finding to elicit discussion of participants' understanding and reactions. VUS information was not prominent in the company report and did not include variant level details, so many participants did not recognize they had received a VUS unless their attention was specifically directed to it. Each interview was conducted in a private space over Zoom, lasted approximately 45 to 60 min, and was audio‐recorded and transcribed for accuracy. Transcripts were not returned to study participants, no repeat interviews were conducted, and no field notes were made during or after the interview by the interviewer. Participants were compensated with a $100 electronic gift card for their time. Those who completed a qualitative interview were also invited to complete a follow‐up survey collecting additional personal and family health history information and exploring their engagement with genetic counseling after undergoing PM‐GT. Twenty‐nine participants completed a follow‐up survey between December 2024 and January 2025. Only data from the 29 participants who completed both the interview and the survey are included in this analysis. The survey consisted of eight questions about participants' personal and family health history and 10 open‐ended questions about their experience with genetic counseling. Participants were compensated with a $25 electronic gift card for completing the survey.

### 2.3 Data Analysis

We adopted a pragmatic orientation, prioritizing methods that would best address our research questions about consumers' experiences with genetic counseling in the PM‐GT setting. Within this approach, we used qualitative inductive content analysis to identify patterns and themes across interviews and survey responses and quantitative content analysis to quantify the frequency of coded responses across participants (Elo and Kyngäs 2008; Ramanadhan et al. 2021; Rourke and Anderson 2004). Only themes and quotes generated from the survey portion of the study were included in this manuscript.

Two members of the research team independently reviewed a subset of qualitative interviews to develop a preliminary coding schema (Authors KL and MP). A third research team member then refined and consolidated these into a codebook (Author MK). As new concepts and themes emerged, the codebook was refined accordingly. The same process was then repeated for the follow‐up surveys.

Interview transcripts and follow‐up surveys were independently coded by two research team members using Dedoose software. Responses could be assigned to multiple thematic or conceptual codes. Frequency counts are reported in Tables 1, 2, 3, 4. Responses mentioned by only one participant were described in the text only if they were not included in a table. In cases of intercoder disagreement, the research team met to resolve discrepancies, ensuring that the final codes reflected a shared interpretation of the data rather than a single individual's perspective. Thematic saturation for the interviews was reached after coding approximately 10 interviews, and thematic saturation for the follow‐up surveys was reached after coding approximately 10 surveys. Participants did not provide feedback on the findings. To ensure reliability and provide transparency about coding consistency, intercoder agreement was calculated using Cohen's kappa statistic for a subset of transcripts. The kappa statistic measures the extent to which agreement between coders exceeds what would be expected by chance and was calculated to be 0.96. A kappa coefficient (*κ*) of 0.8 served as the threshold for adequate agreement among coders (McHugh 2012). Although consensus, rather than statistical agreement, determined the final coding, we include the kappa statistic as a descriptive indicator of the extent to which coders initially applied the coding scheme in a consistent manner. This combination of consensus coding and the kappa statistic reflects a pragmatic orientation that emphasizes collaborative interpretation while also providing a quantitative check on our coding framework. We have endeavored to report this study in accordance with the COREQ (**CO**nsolidated criteria for **RE**porting **Q**ualitative research) guidelines (Tong et al. 2007).

**TABLE 1:** Participant demographics.

Characteristic	Value
Age (years)	
Mean	52
Range	21–75
Sex, no. (%)	
Female	18 (62%)
Male	11 (38%)
Race, no. (%)	
Asian	3 (10%)
Black	2 (7%)
White	24 (83%)
Ethnicity, no. (%)	
Hispanic/Latino	2 (7%)
Non‐Hispanic	26 (90%)
Unknown	1 (3%)
Yearly household income, no. (%)	
Less than $20,000	1 (3%)
$20,000–34,999	2 (7%)
$35,000–49,999	2 (7%)
$50,000–74,999	5 (17%)
$75,000–99,999	6 (21%)
$100,000–149,999	5 (17%)
$150,000–199,999	2 (7%)
$200,000–249,999	3 (10%)
$250,000 or more	3 (10%)
Education, no. (%)	
Trade school	3 (10%)
Some college	3 (10%)
Bachelor's degree	6 (21%)
Master's, professional, or doctoral degree	17 (59%)
Marital status, no. (%)	
Single, never married	8 (28%)
Currently married	14 (48%)
Domestic partnership	3 (10%)
Separated	1 (3%)
Divorced	2 (7%)
Widowed	1 (3%)
Personal history of disease, no. (%)	
Cancer	3 (10%)
Cardiovascular	5 (17%)
Family history of disease, no. (%)	
Cancer	24 (83%)
Cardiovascular	21 (72%)

## 3 Results

### 3.1 Participant Characteristics

**FIGURE 1:**
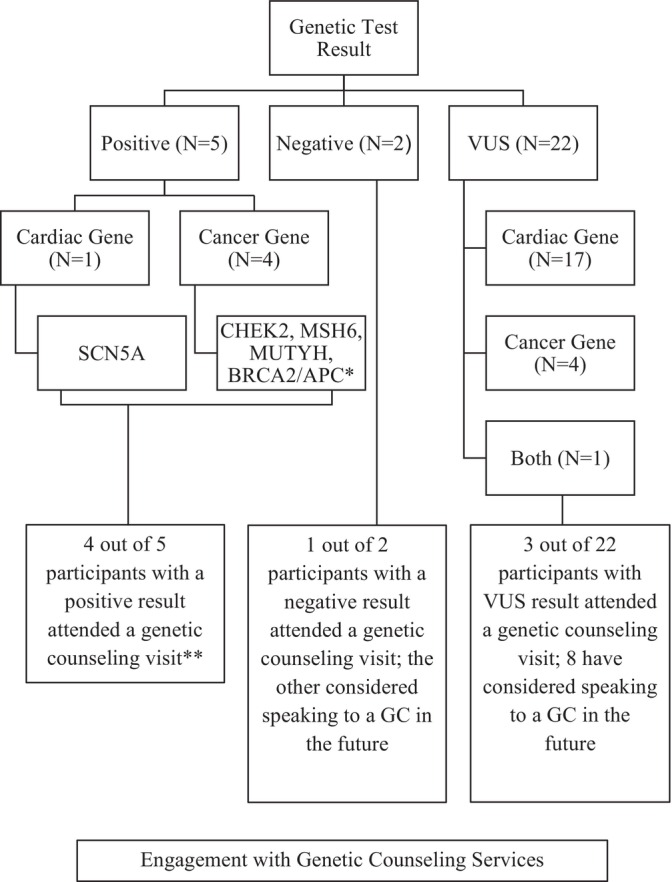
Participant PM‐GT results and engagement with genetic counseling services. *One participant was identified as having pathogenic variants in both the *BRCA2* and *APC* genes. **One participant with a positive result maintained that they did not speak with a GC, despite Color Health requiring all participants with positive results to undergo genetic counseling before their results were released.

Of the 31 ENLITE Study participants who took part in a qualitative interview, 29 also completed a follow‐up survey about genetic counseling in PM‐GT. Participants had a mean age of 52 years and were predominantly female (62%), white (83%), and non‐Hispanic (90%). Most reported a household income above $75,000 (65%), held a bachelor's degree or higher (80%), and were married or in a domestic partnership (58%). Ten percent had a personal history of cancer, 17% had a personal history of cardiovascular disease, 83% had a family history of cancer, and 72% had a family history of cardiovascular disease (Table 1). With respect to their PM‐GT results, five participants received positive findings, 22 received a VUS result, and two received negative results (Figure 1).

### 3.2 Interaction With Genetic Counseling Services

Participants were asked whether they spoke with a GC at any point during the testing process. None reported speaking with a GC prior to purchasing PM‐GT. Eight participants spoke with a Color Health‐affiliated GC after receiving their results. Among them, four had positive results, three had VUS results, and one had negative results. Notably, one participant with a positive result maintained that they had not spoken with a GC, even though Color Health requires all participants with positive results to complete genetic counseling before their results are released. No participants reported speaking to a GC outside of Color Health after receiving their results. Of the remaining 20 participants who responded to this question and had not yet spoken with a GC, nine (45%) indicated that they were considering doing so in the future. Of these nine participants, eight had a VUS result and one had a negative result (Figure 1).

### 3.3 Factors That Influenced Consumer Engagement With Genetic Counseling

Participants were invited to share their thoughts on reasons to pursue—or not pursue—genetic counseling after receiving PM‐GT results (Table 2). Among those who engaged with genetic counseling, one of the most cited reasons was a desire for more information to better understand their results (*n* = 6).

I got unexpected results and wanted to know more information. I like having information on health‐related things. (52‐year‐old Female, Cancer VUS)

Additional reasons for engaging with genetic counseling included having a family history of disease (*n* = 3) and general curiosity about their personal health (*n* = 3).

**TABLE 2:** Reasons for engagement with genetic counseling following receipt of PM‐GT results.

Please describe why you decided to pursue genetic counseling or not	Frequency counts
**Why**	
Wanted more information/to better understand results	6
Family history of disease	3
Curious about personal health	3
Results spurred investigation	2
Could not access results without speaking to a GC	1
Benefit of participating in the research study	1
To help primary care physician understand how to use results	1
To be informed about scientific advancement/updates since study participation	1
**Why not**	
Negative results/no detected mutations	7
Not necessary/not worthwhile	5
Not helpful	3
Did not have any questions/concerns	2
Would rather not know more information about results	1
Too expensive/cost‐related concerns	1
Did not have time	1
Have not gotten around to it	1
Does not change lifestyle or medical management	1
Medical management (e.g., screening) is limited or unavailable	1
Not urgent	1
**Other**	
Did not pursue counseling after testing, but is now considering it	1
Anticipates having questions at some point	1

For participants who did not engage with genetic counseling, the most frequently cited reason was receiving a result that did not identify a pathogenic variant (*n* = 7).

Color results indicated that there were no presently known signifiers of genetic conditions, so I didn't feel like it was urgent, but I anticipate that I'll have questions at some point. (47‐year‐old Female, Cancer VUS)

Other reasons for not engaging with genetic counseling included the perception that genetic counseling was unnecessary or not worthwhile (*n* = 5), the belief that genetic counseling would not be helpful (*n* = 3), or not having any remaining questions or concerns (*n* = 2).

### 3.4 Barriers to Pursuing Genetic Counseling Services

Participants were invited to share whether they would have liked to attend a genetic counseling session but were unable to do so. Most reported that they did not encounter any barriers to accessing genetic counseling services (*n* = 19). Among those who did encounter barriers, the most common issue was being too busy or believing that genetic counseling would be too time‐consuming (*n* = 5). Other barriers included personal life circumstances, such as getting married, buying a house, or caregiving (*n* = 2).

I married, moved to a different state and [am] now renovating [a] house. Have been running around like [a] chicken without a head. Never found time. (63‐year‐old Female, Cardiac VUS)

A couple of participants indicated that they were not interested in genetic counseling or felt that it was unnecessary (*n* = 2), while others noted that it simply was not a priority (*n* = 2). Additional barriers included losing contact information for a Color Health GC (*n* = 1), or forgetting to reach out to initiate contact with a GC (*n* = 1). One participant reported that, despite the availability of telehealth genetic counseling services, living in a rural community with limited cellular service and broadband internet access, combined with an inability to travel to an in‐person appointment, was a barrier to accessing care.

### 3.5 Importance of Genetic Counseling in the PM‐GT Experience

Participants were prompted to describe whether they felt that genetic counseling was important to their PM‐GT experience, and to what extent (Table 3). Among those who commented on its importance, genetic counseling was most often described as important for understanding PM‐GT results, particularly when results were complex (*n* = 7). Of these seven participants, two had a positive result, four had a VUS result, and one had a negative result.

[Genetic counseling] was [important] because without it, I don't think I would have understood the results at all. (33‐year‐old Female, Cardiac Positive, *SCN5A*)

Participants also highlighted the importance of genetic counseling for understanding disease risk (*n* = 4), as well as informing medical management decisions and exploring next steps and (*n* = 3).

[Genetic counseling] was [a] very important part. It let me know what my risks are and what my next steps should be. Getting a test, but not interpreting the results means nothing. (52‐year‐old Female, Cancer VUS)

For participants who felt that genetic counseling was not important, the most common reasons were that it seemed unnecessary (*n* = 3) or that they already understood their results (*n* = 2).

I think I understood the results from the written reports I got back, didn't really need verbal counseling. (66‐year‐old Male, Cancer Positive, *CHEK2*)

Some participants noted that while they did not pursue genetic counseling, they would have sought it had their results indicated a genetic risk (*n* = 4).

If there was a risk of cancer, I would seek the advice of a genetic counselor. (56‐year‐old Male, Cardiac VUS)

**TABLE 3:** Opinions regarding importance of genetic counseling to PM‐GT experience.

Please describe whether, and to what extent, you feel that genetic counseling was an important part of your genetic testing experience	Frequency counts
**Important**	
To understand results, especially if they are complex	7
To be informed about disease risk	4
To be informed about next steps/medical management	3
To answer questions	1
Considered GC to be a large part of testing experience	1
Empowerment/confidence	1
Trusted expert explanations	1
Positive experience	1
Post‐test counseling is important	1
**Not Important**	
Felt unnecessary	3
Understood results	2
Would not change lifestyle or medical management	1
**Other**	
Would have sought counseling if they had genetic risk or results were different	4
Ambivalent/cannot say whether it is important or not	4
Was not sure/cannot remember	2
Important, but time consuming/has not had the time to reach out	1

### 3.6 Advantages and Disadvantages of Genetic Counseling

Participants were asked to reflect on the advantages and disadvantages they considered when deciding whether to pursue genetic counseling after receiving their PM‐GT results (Table 4). More participants commented on advantages than on disadvantages. The most discussed advantage was that genetic counseling was informative and helped strengthen participants' understanding of their results, particularly their disease risk (*n* = 11).

Just to have the clarification of the results and have further understanding. I feel that was an advantage. (44‐year‐old Female, Cardiac VUS)

Additional advantages included the opportunity to discuss next steps for medical management and take action based on results (*n* = 7), the potential for positive mental health outcomes such as relief or peace of mind (*n* = 3), and the opportunity to have questions answered by a GC (*n* = 2).

**TABLE 4:** Advantages and disadvantages of genetic counseling.

Please describe the advantages and disadvantages you considered when deciding whether to speak with a genetic counselor about your test results	Frequency counts
**Advantages**	
Informative, strengthened understanding, including of disease risk	11
Opportunity to discuss next steps/medical management/can take action/opportunity to catch disease early or receive preventative care	7
Positive mental health implications (e.g., relief/peace of mind)	3
Can have questions answered	2
Better understanding of disease risk for relatives	1
Provide guidance/opportunity to talk with a counselor	1
Learn about Color's process for recontacting customers regarding VUS reclassification	1
**Disadvantages**	
Negative mental health implications (e.g., fear, denial, stress)	6
Not helpful/useful	4
Not planning on having children	1
Did not have any questions	1
Results will not change screening/medical management	1
Time consuming/too much information to cover	2
Conversation would be too high‐level for a lay person to understand	1
**Other**	
Did not comment on advantages/disadvantages	3
Would have been more useful if results were different (positive/concerning/high risk) or they could lead to actionable information	1
Did not consider counseling	1

In contrast, the most noted disadvantage of genetic counseling was the concern that it could be associated with negative mental health effects, such as fear, denial, or stress (*n* = 6).

The results might be scarier than expected. Most people like to ignore items and only deal with problems when [it is] too late to do anything. (58‐year‐old Male, Cancer Positive, *MUTYH*)

Other cited disadvantages included the belief that genetic counseling would not be helpful or useful (*n* = 4) and the perception that the process would be too time‐consuming (*n* = 4).

I didn't really see any advantages for me personally in speaking to a genetic counselor, especially considering I have not and will not be having children myself. (41‐year‐old Female, Negative)

### 3.7 Specific Experiences That Influenced Engagement With Genetic Counseling

Participants shared whether there was a specific experience during the testing process or after receiving their results that influenced their engagement with genetic counseling. The majority reported that they had no such experience (*n* = 19). Among those who did, the most common experience was difficulty interpreting their results on their own (*n* = 2).

Attempting to read and fully understand my results made me want to seek counseling. (46‐year‐old Male, Negative)

Other influential experiences included receiving unexpected results (*n* = 1), concerns about disease risk (*n* = 1), and the belief that genetic counseling was a standard part of the PM‐GT process (*n* = 1).

For participants who shared a specific experience that led them to forgo genetic counseling, the most common was receiving negative or non‐concerning results (*n* = 4).

Seeing that there were no big red flags for cancer or heart disease made it feel less urgent to speak with someone. (47‐year‐old Female, Cancer VUS)

One participant also noted that the written results provided sufficient explanation, making counseling unnecessary (*n* = 1).

### 3.8 Confidence in the Genetic Counseling Across Required and Optional Pathways

Participants were asked to describe their confidence in their decision to pursue or decline genetic counseling. However, counseling pathways varied by result type. Participants with positive results were required to complete genetic counseling before receiving their results, whereas those with negative or VUS results could choose whether to pursue counseling after results disclosure. Accordingly, responses reflect confidence in counseling‐related choices and experiences within these different pathways. Most participants expressed confidence in their decision, regardless of pathway (*n* = 22). Among those who described confidence in undergoing genetic counseling, the most cited reason was that it helped them understand their PM‐GT results (*n* = 3). One participant, in particular, felt that testing was pointless without genetic counseling.

Why get tested if you don't want counseling. I have a PhD in biology and know a [fair] bit about this stuff, but I don't know everything I need to know in order to make the best decisions for me and my kids. If I got something really bad, they need to know their risk also. (70‐year‐old Male, Cancer Positive, *BRCA2* and *APC*)

Participants also mentioned gaining valuable information about personal disease risk (*n* = 2), clarifying disease risk for relatives (*n* = 1), and satisfying general curiosity (*n* = 1).

I feel grateful to understand what my results meant for me and how to read them. (33‐year‐old Female, Cardiac Positive, *SCN5A*)

Among participants who did not pursue genetic counseling after receiving their PM‐GT results (i.e., those in the optional counseling pathway), confidence in that choice was most often attributed to concerns that counseling would be too time‐consuming (*n* = 2) and to the belief that it was unnecessary given negative or non‐concerning results (*n* = 2).

Right now I feel the decision not to pursue counseling was right for me because I do not wish to spend the time and money to have the results examined in greater depth. (41‐year‐old Female, Negative)

Other considerations included concerns about the cost of counseling services (*n* = 1), a perceived lack of immediate need for counseling (*n* = 1), and the belief that there would be no changes to medical management based on the results (*n* = 1). However, some participants expressed lower confidence (*n* = 2) or ambivalence (*n* = 1) about their counseling‐related decision or experience. Several others commented that they plan to—or would like to—pursue counseling in the future (*n* = 4).

I wanted to understand what my findings meant. I didn't receive one‐on‐one counseling, but would like to know more. (57‐year‐old Female, Cardiac VUS)

## 4 Discussion

Although prior work has focused on the traditional DTC setting, this study explored how consumers engaged with genetic counseling in the context of physician‐mediated genetic testing (PM‐GT), focusing on their use of counseling services, the factors that influenced their engagement with counseling, and their perceptions of its value (Bloss et al. 2013; Carere et al. 2016; Kilbride et al. 2024; Koeller et al. 2017; Marzulla et al. 2021).

Many PM‐GT consumers were interested in genetic counseling. However, actual uptake of counseling was relatively low; none of the participants consulted with a genetic counselor (GC) before testing and only eight did so after receiving their results. While the lack of pre‐test engagement likely reflected Color Health's model, which does not offer individualized pre‐test counseling, the low uptake of post‐test counseling suggests that most consumers did not take advantage of the counseling that was available to them. Notably, nearly half of those who did not speak with a GC expressed interest in doing so in the future, indicating that many individuals who receive negative or VUS results still perceive value in counseling. Compared to prior research on traditional DTC genetic testing that reported low engagement with genetic counseling services, participants in this study showed greater interest in and uptake of counseling (Bloss et al. 2013; Kaufman et al. 2012; Koeller et al. 2017). This may reflect growing public awareness of genetic counseling and a perception that the greater complexity and scope of PM‐GT panels make professional support valuable for interpreting results. However, it is worth emphasizing that more than half of the participants who spoke with a GC had received positive test results. Because Color Health requires all individuals with positive findings to consult with a GC before accessing their results through the online portal, most participants who spoke with a GC did so as a required step in receiving their results, rather than by independent choice. As such, only a small number of participants engaged with counseling on their own initiative. Requiring genetic counseling for individuals with positive results, while making it optional for those with negative or VUS results, may have affected how consumers engaged with and perceived genetic counseling. Future research should consider examining whether individuals who receive positive results voluntarily pursue genetic counseling and opt to have their results delivered by a GC even when it is not required.

The gap between expressed interest and actual uptake may also reflect practical barriers that impede access to genetic counseling services. Time constraints were described as the most significant barrier to accessing care, with participants reporting that they were “too busy” or that genetic counseling was “too time‐consuming” amid competing life priorities. These findings suggest that consumers may have misconceptions about the time commitment required for genetic counseling sessions, which typically last 30–60 min and are increasingly available through telehealth platforms, reducing travel burdens and scheduling constraints (Osborne et al. 2025). These misconceptions about time commitment underscore the need for clear pre‐test communication about the availability, accessibility, and duration of these services. Educating consumers upfront that counseling is available remotely and requires a relatively modest time investment, while emphasizing its importance for all result types, could help address this barrier.

Some participants expressed concern about negative mental health effects, which were cited as the greatest disadvantage of genetic counseling services. Because genetic counselors receive extensive psychosocial training to address such concerns, this finding further suggests a gap in PM‐GT consumers' knowledge about what GC services entail, which could be addressed through more comprehensive pre‐test education (Biesecker 2020).

Participants expressed a desire to understand their results, which was the most common motivation for pursuing genetic counseling. Many participants also recognized the value of counseling for clarifying disease risk and identifying appropriate steps for medical management. This emphasis on understanding results is consistent with findings from prior studies on traditional DTC genetic testing, which show that consumers often seek genetic counseling due to personal or family history of disease, uncertainty about their results, or a desire for additional information (Koeller et al. 2017; Marzulla et al. 2021). These patterns suggest that, across both traditional DTC and PM‐GT settings, consumers are motivated to understand their results more deeply, beyond the information that is provided in the test report alone.

The motivation to better understand one's results highlights the broader benefits that genetic counseling may offer. Counseling has been shown to increase knowledge, improve perceived control over one's health, promote positive health behaviors, and enhance the accuracy of risk perception (Madlensky et al. 2017). Determining whether these benefits extend to PM‐GT users could help build the case for more fully integrating genetic counseling into this model. To explore this further, future research should compare the accuracy of consumers' interpretation of their results with and without post‐test counseling to assess the added value of professional support, particularly in cases involving a family history of disease or negative or VUS results.

Although the majority of participants expressed interest in genetic counseling services, many who received negative or VUS results felt that genetic counseling was unnecessary. This perception may reflect a broader belief that counseling is only needed when a pathogenic variant is identified (Sutton et al. 2020). However, clinical guidelines recommend post‐test counseling for individuals who receive negative results. When a family has a strong history of disease but no known familial pathogenic variant, a negative result does not necessarily mean that an individual has average risk. Risk may still be elevated based on family history alone (ACOG Committee on Genetics 2017). Similarly, for individuals without a personal or family history of disease, negative results do not indicate below‐average risk but rather suggest that these individuals remain at approximately the same risk as the general population.

While some individuals undergoing PM‐GT meet professional guidelines for diagnostic testing, PM‐GT is offered on an elective basis, meaning that individuals may pursue testing even if they do not meet those criteria. In our sample, most interviewees who received a VUS also reported a family history of cancer or cardiovascular disease, making clinical context especially important. The American College of Medical Genetics (ACMG) guidelines state that VUS results should not be used to guide clinical decision‐making. At the same time, these guidelines emphasize that screening decisions should be based on clinical indication, including an individual's personal and family history, while laboratories continue to update variant classifications (Richards et al. 2015). Prior research has shown that individuals often struggle to interpret the implications of VUS results on their own, highlighting the need for professional guidance (Mighton et al. 2021; Vears et al. 2020). GCs play an important role in interpreting and contextualizing VUS results and in guiding medical management, but current PM‐GT practices may limit access to this support.

In our study, none of the participants with VUS results were aware before the interview that a VUS had been identified. Because they were informed during the interview, the interview itself may have influenced their subsequent views of genetic counseling and their stated interest in pursuing it. Although Color Health's results reports do indicate whether a VUS was identified in a cancer or cardiovascular disease risk gene, this information appears in a less prominent section of the report. Importantly, the specific gene and variant are not disclosed, and the top of the report states that no mutations were identified, effectively presenting the VUS as a negative test result. If test‐takers want more information about the VUS, they must proactively contact the company to request further details. While not standard across all PM‐GT laboratories, this practice may limit opportunities for appropriate follow‐up, particularly for those who may require additional medical management based on family history alone, or those whose VUS finding may later be reclassified (Chang et al. 2023; Kilbride and Bradbury 2020).

Currently, the National Society of Genetic Counselors (NSGC) recommends that, in the context of elective genetic testing (EGT), VUS results should not be reported and that individuals undergoing EGT should receive both pre‐ and post‐test genetic counseling, regardless of result type (Blout Zawatsky et al. 2023). Accordingly, our findings suggest that current PM‐GT practices may diverge from existing professional recommendations regarding EGT in several important ways. First, VUS results are returned to consumers, a practice that is especially concerning given the lack of context or support needed to make those results meaningful for medical decision‐making. Second, pre‐test counseling is not offered. Third, post‐test counseling is available but underutilized, potentially because it is not the default for negative and VUS results and because test‐takers, particularly those receiving these results, may perceive it as having limited value. More robust genetic counseling—both pre‐ and post‐test—could help address these concerns. Pre‐test counseling could clarify the range of possible results, explain their potential health implications, and help individuals understand how personal and family history may influence future medical decisions. Post‐test counseling could then support consumers in interpreting ambiguous or unexpected findings, reduce the risk of misunderstanding, and guide appropriate follow‐up care. Expanding access to these services, however, would likely require changes to how PM‐GT is delivered and would need to be balanced against concerns about cost and the potential for new barriers to access, especially for individuals who might turn to PM‐GT because they lack access to clinical genetic services.

### 4.1 Study Limitations

This study had several limitations. First, a significant limitation is that VUS results were disclosed to participants during the research interview rather than through standard Color reporting. This disclosure represents an active intervention that could have influenced participants' subsequent decisions about pursuing genetic counseling and their perceptions of its value. Accordingly, the genetic counseling uptake rates and attitudes among VUS participants in this study may not reflect typical consumer behavior for individuals receiving similar results through standard Color Health reporting, where VUS information is not prominent. Participants were not systematically asked whether learning of their VUS during the interview influenced their genetic counseling decisions. As such, we cannot determine how much VUS disclosure influenced subsequent engagement with genetic counseling. Future research should examine genetic counseling decision‐making among VUS recipients who learn of these results through standard reporting mechanisms without research‐based disclosure. Second, there was potential for recruitment bias. Participants were recruited through Research Match, a national volunteer registry, and may reflect individuals who are more interested in research, more knowledgeable about health topics, and more positively inclined toward genetic testing and counseling than the general population. Third, most participants had either positive or VUS results due to intentional oversampling by result type during the qualitative interview phase of the ENLITE Study. As a result, perspectives from individuals with positive or VUS findings may be more prominent in our sample, while perspectives from those with negative results—who make up the majority of PM‐GT users—are less represented. Fourth, the sample size of this study was demographically fairly homogeneous, further limiting generalizability to more diverse populations. Fifth, a time gap of approximately 1.5 years between when participants received their PM‐GT results and when they completed the follow‐up survey may have affected the accuracy of their recollections. Finally, these findings reflect the PM‐GT testing landscape at the time the study was conducted, as the market and available testing offerings may change over time.

## 5 Conclusions

This study highlights a gap between consumers' interest in genetic counseling and their actual use of it in the context of PM‐GT. While uptake was low, many participants who had not spoken with a genetic counselor expressed interest in doing so in the future, suggesting that consumers value having counseling as an option even if they do not act on it immediately. Participants also emphasized the importance of understanding their results, where those who pursued counseling often did so to gain clarity, particularly about disease risk and next steps for medical management. Others, especially those who received negative or VUS results, perceived counseling as unnecessary. This perception raises the possibility that consumers may not fully appreciate the importance of post‐test counseling, which plays a critical role in preventing misinterpretation and ensuring that genetic test results are understood within the context of personal and family health history to guide appropriate follow‐up care.

These findings have several important implications for genetic counseling. First, they suggest that more proactive engagement strategies may be needed, as many consumers who might benefit from counseling, particularly those with negative or uncertain results, do not pursue it on their own. To address this, genetic counseling could be more fully integrated into the PM‐GT process and offered by default, rather than remaining a service that most consumers must actively request. Second, they underscore the importance of clearly communicating the value of genetic counseling across all result types—both before and after testing—to encourage uptake and dispel the misconception that it is only necessary when a pathogenic variant is identified.

As PM‐GT becomes more widely used, it is important to understand how consumers engage with genetic counseling, including why some choose not to use it and how they perceive its value. These insights can help identify shortcomings in current practices and guide changes that better support test‐takers, minimize harm, and promote the responsible use of PM‐GT to improve health outcomes.

## Author Contributions

**Katherine A. Lauro:** conceptualization, data curation, formal analysis, funding acquisition, investigation, writing – original draft, writing – review and editing. **Bob Wong:** data curation, formal analysis. **Jason Iuliano:** conceptualization, writing – review and editing. **Margaret Peo:** data curation. **Jacqueline Park:** data curation, investigation. **Karin M. Dent:** conceptualization, supervision, writing – review and editing. **Angela R. Bradbury:** conceptualization, writing – review and editing. **Madison K. Kilbride:** conceptualization, data curation, formal analysis, funding acquisition, investigation, supervision, writing – review and editing.

## Funding

This study was supported by NIH grant K01 HG010903 (Kilbride). The follow‐up survey was funded by the University of Utah Graduate Program in Genetic Counseling (UUGPGC).

## Disclosure

Artificial intelligence tools were not used in the preparation of this manuscript, consistent with the journal's guidelines.

## Ethics Statement

This study was approved by the University of Utah Institutional Review Board (IRB_00147342).

## Consent

Written informed consent was obtained at the beginning of the ENLITE Study, and participants provided additional consent for the qualitative interview and follow‐up survey described in this manuscript. No non‐human animal research was performed as part of this study.

## Conflicts of Interest

Dr. Bradbury reports receiving research funding from AstraZeneca and Merck for research unrelated to this study. All other authors declare that they do not have any conflicts of interest to disclose.

## Data Availability

Data from this study is available from the corresponding author upon reasonable request.

## References

[jgc470257-bib-0001] 23andMe . n.d. “Do You Speak BRCA?” https://www.23andme.com/brca/?srsltid=AfmBOor‐C06O_FccLRCdATAaCbVjOtUwQ0zWAPuItA9JGTzLNCFvUZaP.

[jgc470257-bib-0002] ACMG Board of Directors . 2016. “Direct‐to‐Consumer Genetic Testing: A Revised Position Statement of the American College of Medical Genetics and Genomics.” Genetics in Medicine 18, no. 2: 207–208. 26681314 10.1038/gim.2015.190

[jgc470257-bib-0003] ACOG Committee on Genetics . 2017. “Committee Opinion No. 693: Counseling About Genetic Testing and Communication of Genetic Test Results.” Obstetrics & Gynecology 129, no. 4: e96–e101. 10.1097/aog.0000000000002020. 28333821

[jgc470257-bib-0004] Bellcross, C. A. , P. Z. Page , and D. Meaney‐Delman . 2012. “Direct‐to‐Consumer Personal Genome Testing and Cancer Risk Prediction.” Cancer Journal 18, no. 4: 293–302. 22846729 10.1097/PPO.0b013e3182610e38

[jgc470257-bib-0005] Biesecker, B. 2020. “Genetic Counseling and the Central Tenets of Practice.” Cold Spring Harbor Perspectives in Medicine 10, no. 3: a038968. 31570379 10.1101/cshperspect.a038968 PMC7050579

[jgc470257-bib-0006] Bloss, C. S. , N. E. Wineinger , B. F. Darst , N. J. Schork , and E. J. Topol . 2013. “Impact of Direct‐to‐Consumer Genomic Testing at Long Term Follow‐Up.” Journal of Medical Genetics 50, no. 6: 393–400. 23559530 10.1136/jmedgenet-2012-101207

[jgc470257-bib-0007] Blout Zawatsky, C. L. , D. Bick , L. Bier , et al. 2023. “Elective Genomic Testing: Practice Resource of the National Society of Genetic Counselors.” Journal of Genetic Counseling 32, no. 2: 281–299. 36597794 10.1002/jgc4.1654

[jgc470257-bib-0008] Carere, D. A. , P. Kraft , K. A. Kaphingst , J. S. Roberts , and R. C. Green . 2016. “Consumers Report Lower Confidence in Their Genetics Knowledge Following Direct‐to‐Consumer Personal Genomic Testing.” Genetics in Medicine 18, no. 1: 65–72. 25812042 10.1038/gim.2015.34 PMC4583799

[jgc470257-bib-0009] Chang, E. Y. , I. Solomon , J. O. Culver , et al. 2023. “Clinical and Laboratory Genetic Counselor Attitudes on the Reporting of Variants of Uncertain Significance for Multigene Cancer Panels.” Journal of Genetic Counseling 32, no. 3: 706–716. 36747331 10.1002/jgc4.1680

[jgc470257-bib-0010] Color Health . n.d. https://web.archive.org/web/20250929145509/https://www.color.com/individuals‐genomics.

[jgc470257-bib-0011] Elo, S. , and H. Kyngäs . 2008. “The Qualitative Content Analysis Process.” Journal of Advanced Nursing 62, no. 1: 107–115. 18352969 10.1111/j.1365-2648.2007.04569.x

[jgc470257-bib-0012] Harris, P. A. , K. W. Scott , L. Lebo , N. Hassan , C. Lightner , and J. Pulley . 2012. “ResearchMatch: A National Registry to Recruit Volunteers for Clinical Research.” Academic Medicine 87, no. 1: 66–73. 10.1097/ACM.0b013e31823ab7d2. 22104055 PMC3688834

[jgc470257-bib-0013] Henry, T. A. 2021. Protect Sensitive Individual Data at Risk From DTC Genetic Tests. American Medical Association.

[jgc470257-bib-0014] Jiang, S. , L. Liberti , and D. Lebo . 2023. “Direct‐to‐Consumer Genetic Testing: A Comprehensive Review.” Therapeutic Innovation & Regulatory Science 57, no. 6: 1190–1198. 37589855 10.1007/s43441-023-00567-5

[jgc470257-bib-0015] Kaufman, D. J. , J. M. Bollinger , R. L. Dvoskin , and J. A. Scott . 2012. “Risky Business: Risk Perception and the Use of Medical Services Among Customers of DTC Personal Genetic Testing.” Journal of Genetic Counseling 21: 413–422. 22278220 10.1007/s10897-012-9483-0

[jgc470257-bib-0016] Kilbride, M. K. , and A. R. Bradbury . 2020. “Evaluating Web‐Based Direct‐to‐Consumer Genetic Tests for Cancer Susceptibility.” JCO Precision Oncology 4: 161–169. 10.1200/PO.19.00317 PMC8714633 34970636

[jgc470257-bib-0017] Kilbride, M. K. , L. J. Kessler , B. Cronier , et al. 2024. “Test‐Takers' Perspectives on Consumer Genetic Testing for Hereditary Cancer Risk.” Frontiers in Genetics 15: 1374602. 39050249 10.3389/fgene.2024.1374602 PMC11266061

[jgc470257-bib-0018] Koeller, D. R. , W. R. Uhlmann , D. A. Carere , R. C. Green , J. S. Roberts , and Group, P. S . 2017. “Utilization of Genetic Counseling After Direct‐to‐Consumer Genetic Testing: Findings From the Impact of Personal Genomics (PGen) Study.” Journal of Genetic Counseling 26, no. 6: 1270–1279. 28512697 10.1007/s10897-017-0106-7 PMC5673568

[jgc470257-bib-0019] LaDuca, H. , E. C. Polley , A. Yussuf , et al. 2020. “A Clinical Guide to Hereditary Cancer Panel Testing: Evaluation of Gene‐Specific Cancer Associations and Sensitivity of Genetic Testing Criteria in a Cohort of 165,000 High‐Risk Patients.” Genetics in Medicine 22, no. 2: 407–415. 31406321 10.1038/s41436-019-0633-8 PMC7000322

[jgc470257-bib-0020] Madlensky, L. , A. M. Trepanier , D. Cragun , B. Lerner , K. M. Shannon , and H. Zierhut . 2017. “A Rapid Systematic Review of Outcomes Studies in Genetic Counseling.” Journal of Genetic Counseling 26, no. 3: 361–378. 28168332 10.1007/s10897-017-0067-x

[jgc470257-bib-0021] Majumder, M. A. , C. J. Guerrini , and A. L. McGuire . 2021. “Direct‐to‐Consumer Genetic Testing: Value and Risk.” Annual Review of Medicine 72, no. 1: 151–166. 10.1146/annurev-med-070119-114727 32735764

[jgc470257-bib-0022] Marzulla, T. , J. S. Roberts , R. DeVries , D. R. Koeller , R. C. Green , and W. R. Uhlmann . 2021. “Genetic Counseling Following Direct‐to Consumer Genetic Testing: Consumer Perspectives.” Journal of Genetic Counseling 30, no. 1: 329–334. 32648332 10.1002/jgc4.1309

[jgc470257-bib-0023] McHugh, M. L. 2012. “Interrater Reliability: The Kappa Statistic.” Biochemia Medica 22, no. 3: 276–282. 23092060 PMC3900052

[jgc470257-bib-0024] Middleton, A. , Á. Mendes , C. M. Benjamin , and H. C. Howard . 2017. “Direct‐to‐Consumer Genetic Testing: Where and How Does Genetic Counseling Fit?” Personalized Medicine 14, no. 3: 249–257. 29767582 10.2217/pme-2017-0001

[jgc470257-bib-0025] Mighton, C. , S. Shickh , E. Uleryk , P. Pechlivanoglou , and Y. Bombard . 2021. “Clinical and Psychological Outcomes of Receiving a Variant of Uncertain Significance From Multigene Panel Testing or Genomic Sequencing: A Systematic Review and Meta‐Analysis.” Genetics in Medicine 23, no. 1: 22–33. 32921787 10.1038/s41436-020-00957-2

[jgc470257-bib-0026] NSGC . 2019. “At‐Home Genetic Testing Position Statement.” https://www.nsgc.org/Advocacy/Position‐Statements/Position‐Statements/Post/consumer‐initiated‐genetic‐testing‐position‐statement#:~:text=The%20National%20Society%20of%20Genetic,consumer%20initiated%2C%20etc)%20without%20the.

[jgc470257-bib-0027] Osborne, A. , E. M. Bland , C. Diamonstein , and K. Fishler . 2025. “Time Tracking and Comparison of Genetic Counseling Tasks in Inpatient and Outpatient Settings.” Journal of Genetic Counseling 34, no. 2: e1935. 38922772 10.1002/jgc4.1935 PMC11953576

[jgc470257-bib-0028] Phillips, K. A. , J. R. Trosman , and M. P. Douglas . 2019. “Emergence of Hybrid Models of Genetic Testing Beyond Direct‐To‐Consumer or Traditional Labs.” Journal of the American Medical Association 321, no. 24: 2403–2404. 31145414 10.1001/jama.2019.5670 PMC6684382

[jgc470257-bib-0029] Ramanadhan, S. , A. C. Revette , R. M. Lee , and E. L. Aveling . 2021. “Pragmatic Approaches to Analyzing Qualitative Data for Implementation Science: An Introduction.” Implementation Science Communications 2, no. 1: 70. 34187595 10.1186/s43058-021-00174-1 PMC8243847

[jgc470257-bib-0030] Rebbeck, T. R. , T. M. Friebel , E. Friedman , et al. 2018. “Mutational Spectrum in a Worldwide Study of 29,700 Families With BRCA1 or BRCA2 Mutations.” Human Mutation 39, no. 5: 593–620. 29446198 10.1002/humu.23406 PMC5903938

[jgc470257-bib-0031] Richards, S. , N. Aziz , S. Bale , et al. 2015. “Standards and Guidelines for the Interpretation of Sequence Variants: A Joint Consensus Recommendation of the American College of Medical Genetics and Genomics and the Association for Molecular Pathology.” Genetics in Medicine 17, no. 5: 405–423. 25741868 10.1038/gim.2015.30 PMC4544753

[jgc470257-bib-0032] Rourke, L. , and T. Anderson . 2004. “Validity in Quantitative Content Analysis.” Educational Technology Research and Development 52, no. 1: 5–18.

[jgc470257-bib-0033] Shah, P. D. , and S. M. Domchek . 2020. “The Contemporary Landscape of Genetic Testing and Breast Cancer: Emerging Issues.” Breast Journal 26, no. 8: 1549–1555. 32691458 10.1111/tbj.13968

[jgc470257-bib-0034] Sutton, E. J. , A. T. Beck , K. O. Gamm , J. B. McCormick , I. J. Kullo , and R. R. Sharp . 2020. “‘They're Not Going to Do Nothing for Me’: Research Participants' Attitudes Towards Elective Genetic Counseling.” Journal of Personalized Medicine 10, no. 4: 143. 32987879 10.3390/jpm10040143 PMC7711758

[jgc470257-bib-0035] Tong, A. , P. Sainsbury , and J. Craig . 2007. “Consolidated Criteria for Reporting Qualitative Research (COREQ): A 32‐Item Checklist for Interviews and Focus Groups.” International Journal for Quality in Health Care 19, no. 6: 349–357. 17872937 10.1093/intqhc/mzm042

[jgc470257-bib-0036] Truty, R. , J. Paul , M. Kennemer , et al. 2019. “Prevalence and Properties of Intragenic Copy‐Number Variation in Mendelian Disease Genes.” Genetics in Medicine 21, no. 1: 114–123. https://www.gimjournal.org/action/showPdf?pii=S1098360021000988. 29895855 10.1038/s41436-018-0033-5 PMC6752305

[jgc470257-bib-0037] Vears, D. F. , K. Sénécal , and P. Borry . 2020. “Genetic Health Professionals' Experiences Returning Results From Diagnostic Genomic Sequencing to Patients.” Journal of Genetic Counseling 29, no. 5: 807–815. 31856387 10.1002/jgc4.1209

